# Anovestibular fistula with normal anal opening: Is it always congenital?

**DOI:** 10.4103/0971-9261.44764

**Published:** 2008

**Authors:** Prashant Jain, Pankaj Mishra, Hemanshi Shah, Sandesh Parelkar, S. S. Borwankar

**Affiliations:** Department of Pediatric Surgery, KEM Hospital, Parel, Mumbai, India

**Keywords:** Anal fistula, anovestibular fistula, diarrhea, perineal canal

## Abstract

**Aim:**

To review 12 cases of anovestibular fistula with normal anal opening.

**Methods:**

Retrospective analysis of 12 children with anovestibular fistula and normal anal opening were treated between the years 2000 and 2007. Of these, 11 patients were diagnosed as having acquired anovestibular fistula with normal anal opening and were managed by conservative management.

**Results:**

Most of them presented with diarrhea and labial redness. One patient was considered to have fistula of congenital origin and was managed surgically. Eleven patients presented between the ages of 1.5–11 months and were considered as cases of acquired anovestibular fistula and only two of them required surgical management in the form of colostomy and fistula excision. Others were successfully managed by conservative treatment; the fistulous output and labial redness decreased gradually within a period of 5–19 (average 11.5) days.

**Conclusions:**

Not all presentations of anovestibular fistula with normal anal opening can be considered as congenital. Presence of inflammation, paramedian fistula, and a favourable response to conservative management/colostomy suggest acquired etiology. Trial of conservative management should be given in the acquired variety.

## INTRODUCTION

Communication between the anorectum and urogenital tract with normal anal opening may be congenital or acquired. This condition is described in both sexes and has been considered congenital by various authors and has been called as ‘double termination of the alimentary canal’, ‘perineal canal’, and ‘H’ or ‘N’ type fistula. The management proposed in various series for anorectovestibular fistulae with normal anal opening is surgical. We reviewed 12 cases of anovestibular fistula with normal anal opening over a period of eight years and discuss the probable etiology, management, and natural course of the disease.

## PATIENTS AND METHODS

**Figure 1:**
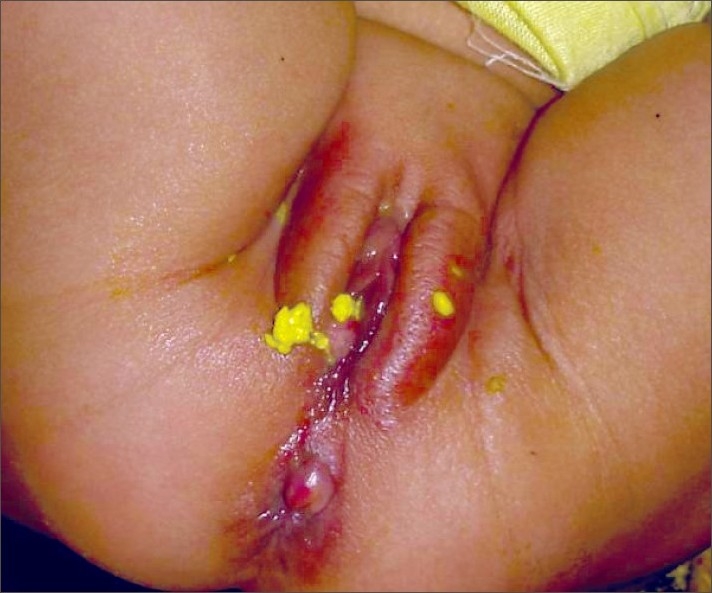
Acquired fistula with labial redness and edema

This is a retrospective review of 12 cases of anovestibular fistula cases with normal anal opening admitted between 2000 and 2007. These cases were reviewed with respect to clinical history, findings, and management. The patients presented between the ages of 1.5–11 months, except for one, an 11-year-old female. They presented with complaints of diarrhea followed by labial redness and edema, and eventually discharge of stools from the vestibular region [Figure 1]. None of the patients had perianal abscess or perianal fistula at presentation. The stool culture was sterile in all patients. Radiological examination was done only in the 11-year-old patient.

**Figure 2:**
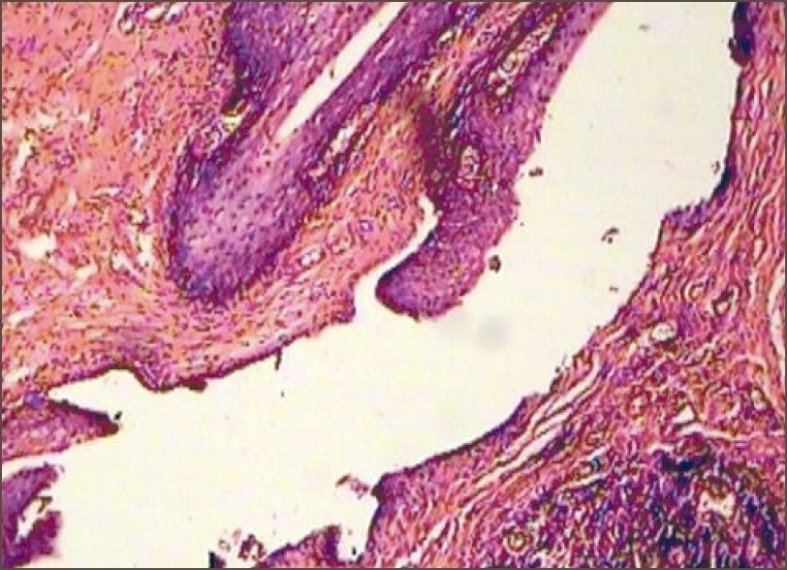
Histopathology of acquired fistula lined by squamous epithelium with inflammation along with rectal mucosa

All the patients, except the 11-year-old girl, were diagnosed with acquired anovestibular fistula secondary to diarrhea and subsequent perineal inflammatory pathology. They were managed conservatively by intestinal antiseptics, oral metronidazole, sitz baths, and gentle anal dilatation (four times a day) for 8–10 days. The patients were observed for the reduction in inflammation and closure of the fistula. Patients who responded to conservative management were examined under general anesthesia to assess complete closure. Patients who did not respond to conservative treatment were managed surgically. The 11-year-old girl with no features of local inflammation at presentation or in the past was diagnosed with congenital fistula and treated surgically by excision of the fistula omit words Figure 2.

## RESULTS

None of the 11 cases presenting between the ages of 1.5–11 months, had any complaints at birth. History of loose stools for 5–10 days was present in all these patients prior to presentation. Three patients had history of swelling at the posterior fourchette which burst open spontaneously followed by purulent and then later fecal discharge. Labial redness and edema was present in nine patients at presentation. Fistulous opening was identified in eight patients from 4 O'clock to 7 O'clock position. Cannulation was possible in five patients. One of the patients had anal stenosis and a ‘cut-back anoplasty’ was performed.

On conservative management, the fistulous output and labial redness decreased gradually within a period of 5–19 (average 11.5) days. A trial of one month was given for spontaneous closure. The patients were examined under general anesthesia and there was no evidence of fistula in nine patients. Two patients who did not respond to the conservative management required surgical procedures. Of these, one patient required colostomy due to recurrence and subsequent fistula excision. The fistula was located at the level of the dentate line. In the other patient, the fistula closed spontaneously following colostomy.

The 11 year old girl who had history of fecal discharge from vestibule since the age of one month was diagnosed to have fistula of congenital origin. She was treated by primary surgical repair. All the other nine patients who were treated by conservative management remained asymptomatic on mean follow up of 4.5 years with no evidence of recurrence.

## DISCUSSION

Anovestibular fistula with normal anus was first described by Bryndorf and Madsen.[1] It has also been called ‘double termination of alimentary tract’,[2] ‘perineal canal’,[3] ‘N’ or ‘H’ type,[4] and ‘congenital anal fistula with normal anus’.[5] The lesion is common in females and is more common in Asian countries.[2,5] The incidence in India and Japan being 3.8% and 7.1%, of all the anorectal malformations, respectively.[2]

A few reports in males have shown that the ‘H’ type of urethroanal fistulae are more commonly associated with congenital anomalies like renal malformations, urethral hypoplasia or atresia, tracheoesophegeal fistula, etc.[5] However in females, congenital anomalies are not common, except for stenotic or anteriorly placed anus[1]

There remains a controversy regarding the etiology and pathogenesis of the condition.[6] Most of the authors consider the condition to be congenital in origin[2] and advise surgical approach for definitive treatment. Brem *et al*,[5] in his report of three cases suggested congenital etiology because of early onset of symptoms and absence of inflammation. In addition, histopathology did not reveal fibrous tracts with granulation tissue. According to him, the congenital nature of the lesion in boys is undisputed.

In addition, the embryologic basis remains speculative and may not be the same in males and females. Stephens[7] has proposed impaired alignment of the two septa dividing internal cloaca as the etiology of the H-type fistula. According to de Vries and Friedland,[4] who described the fistula in males, H-type fistula is persistence of the cloacal duct of Reichel, which links anterior and posterior compartments of the cloaca. The most accepted theory is interruption of the dorsal part of the cloacal membrane by an isolated defect.[3,8]

Because of the association with vulvar abscess, acquired etiology has been suggested. In a series of 12 cases, Tushida *et al*,[9] were reluctant to draw conclusions about the pathogenesis. They concluded that the failure of migration of urorectal and uroanal septum with excessive posterior fusion of the genital folds might cause a patent or a partly patent anorectovestibular fistula despite a normal anal opening. Then, without inflammation in the case of a patent fistula and with secondary inflammation in the case of a partly patent fistula, an abnormal opening or openings may be formed at the vestibule. Presence of local inflammation, multiplicity of abnormal openings, lateral position of the opening in the vestibule were suggestive of acquired disease.

The lining of the fistula in most of the reported cases is squamous (in girls) or transitional (in boys).[10] When the histology of congenital and acquired fistula was compared, in both cases the tract was lined by squamous epithelium and had inflammation. The epithelial lining in acquired fistula could be the result of inflammatory metaplasia as also seen in cases of fistula in ano Fig 2.[11] It is said to be an acquired perineal abscess – fistulization – epithelization sequence.[12]

We believe that the anovestibular fistula with normal anal opening can be of congenital or acquired origin. Most of our patients had no history of passage of stools from the vestibule at birth and presented with history of inflammation and laterally situated fistulous opening. In patients in whom cannulation was possible, the fistulous opening was present just at the dentate line. These factors along with response to conservative management favors an acquired etiology. The inflammation of the crypts of Morgagni may lead to the fistulous communication and has a self-limiting course like fistula in ano.[13] Lateral location of the acquired fistula is similar to perianal abscess and fistula in ano. The anal opening could be stenotic or anteriorly placed.[2,14] In the only patient with congenital anomaly, the stenotic anal opening, could be the cause of cryptits leading to fistula. Except for one, all our cases appeared acquired following cryptitis and responded well to conservative management.

Various surgical approaches like vestibule-anal pull through,[2] pull through of the anterior rectal wall,[10] limited perineal PSARP,[15] endorectal pull through, and direct excision[16] have been described to eradicate the fistula. The procedure can be combined[10] with colostomy. Colostomy alone does not cure the fistula.[13] However, in our series one patient responded to only colostomy suggesting acquired origin.

Presence of inflammation, paramedian fistula, and response to conservative management/colostomy favours acquired etiology in many cases of anovestibular fistula with normal anal opening. These have to be differentiated from fistulae of congenital origin. We recommend that trial of conservative management should be given in cases of acquired fistula.
